# Caffeine intake and awareness of its adverse effects: Insights from the medical students at the University of Tabuk

**DOI:** 10.1371/journal.pone.0335391

**Published:** 2025-10-24

**Authors:** Omnia S. El Seifi, Faten Ezzelarab Younis, Nouf Ali Alsaiari, Rama Mathel Alanazi, Atheer Khalaf Alanazi, Lama Mana Alamri, Mona Salem Albalawi, Shahad Hammad Alatawi, Eman M. Mortada

**Affiliations:** 1 Department of Family and Community Medicine, Faculty of Medicine, University of Tabuk, Tabuk, Saudi Arabia; 2 Sixth-year medical student, Faculty of Medicine, University of Tabuk, Tabuk, Saudi Arabia; 3 Family and Community Medicine Department, College of Medicine, Princess Nourah Bint Abdulrahman University, Riyadh, Saudi Arabia; Sorbonne Paris Nord University, FRANCE

## Abstract

**Background/Objectives:**

Medical students may consume more caffeine-containing beverages to cope with their stressors, resulting in negative effects on physical or mental well-being. This study aimed to determine the prevalence of caffeine use among medical students as well as their awareness of the negative consequences of caffeine use and its implications on mental health.

**Methods:**

305 medical students participated in this cross-sectional study. An online self-administered questionnaire was provided, which included sociodemographic data, caffeine intake patterns, caffeine side effects, and the World Health Organization’s Five Well-Being Index to measure mental health.

**Results:**

81.3% of the medical students reported consuming caffeine. Of them, 52% consumed ≥ 400 mg of caffeine per day despite 73.1% of them being adequately aware of its negative consequences. The percentage of medical students who reported some negative effects from caffeine use was 80.6%. Being male (OR: 10.6) and consuming caffeine for several reasons, such as enhancing academic achievement (OR: 8.4), staying awake (OR: 6.5), as a habit (OR: 4.6), or elevating mood (OR: 3.0), all significantly associated with high caffeine intake. On the other hand, being highly aware of its side effects is protective (OR: 0.69). Furthermore, there was a strong inverse correlation between the mental well-being score and caffeine consumption (r = −0.563, P < 0.001).

**Conclusions:**

Because caffeine consumption is remarkably prevalent among the studied medical students, and one-third of those who consumed a lot of caffeine had poor mental well-being, preventive intervention programs need to be implemented to lower their caffeine consumption.

## Introduction

Caffeine is the most often utilized active ingredient worldwide, and its consumption has grown dramatically in recent years to reach about 80% all over the world [1]. Coffee is the most widely consumed caffeine-containing beverage, with a higher caffeine content than the majority of other beverages, including tea, soft drinks, energy drinks, and chocolate [2].

Caffeine is widely used to improve mental alertness by boosting brain function, wakefulness, productivity, preventing sleep, enjoying the tasty flavor, taking advantage of the social aspects of consumption, enhancing mood, boosting energy, improving performance, and reducing stress [3].

Due to different sensitivities and reactions depending on genetics, age, medical history, and tolerance, it can be difficult to pinpoint an exact caffeine intake for each individual. Research indicates that 6 mg of caffeine/kg of body weight is suitable for people with normal sensitivity to the caffeine molecule, and adults can safely take 400 mg per day, or roughly four or five cups, based on the average human body weight worldwide [4,5]. This is in line with an evaluation carried out by the European Food Safety Authority (EFSA) and the US Food and Drug Administration (FDA) [6,7].

Excess caffeine has a variety of impacts on the cardiovascular, gastrointestinal, respiratory, and renal systems. Aside from slightly increasing systolic and diastolic blood pressure, higher dosages may cause tachycardia and arrhythmias. Additionally, caffeine has a diuretic impact by raising the glomerular filtration rate [8]. Also, it was reported that high caffeine use significantly affects overall sleeping duration and is considered a leading factor for the development of insomnia [9].

Like other psychoactive chemicals, excessive caffeine consumption can result in dependence, with scientific proof that caffeine-withdrawal syndrome exists. Caffeine dependency is recognized as a clinical illness with a prevalence that varies between 9 and 22% by the World Health Organization and the American Psychiatric Association [10].

Students at universities reported utilizing caffeine to improve their mood, performance, or level of alertness. Additionally, university students reported feeling much more awake, focused, alert, and clear-minded after taking small doses of caffeine, which is ideal for their academic work [11,12].

Medical students are known to have stressful lives due to the nature of their study, which necessitates prolonged periods of alertness and intense focus to manage their coursework and tests; this may have a significant effect on their mental well-being and, at the same time, may force them to raise caffeine consumption, especially on exam days [11,12]. Compared to their colleagues of the same age who are not in medical schools, medical students experience higher rates of mental stress, anxiety, and depression [13].

Medical students frequently utilize caffeine as a coping strategy to improve alertness and overcome fatigue, which is usually linked to poor sleep quality and higher academic stress and anxiety. However, because caffeine, especially in high quantities, can exacerbate anxiety and sleep issues and have negative impacts on mental health, its use may create a feedback loop. Caffeine intake and mental well-being are interrelated and influenced by common variables like stress, exhaustion, perceived academic load, and poor sleep quality [14].

Many studies exploring caffeine consumption among university students show that about 90% of US university students drank caffeine-containing beverages [11], and other countries, including Australia, Canada, and Italy, have the same pattern [15].

In the Middle East and North Africa (MENA) region, there is an increase in the prevalence of caffeine consumption in different forms among the general population as well as adolescents (12–18 years) and young adults (18–35 years), particularly [2]. Saudi Arabia ranks among the top ten nations in coffee consumption. The Kingdom imports between 70,000 and 90,000 tons of coffee annually, and Saudis spend over Saudi Arabian Riyal (SAR) 1 billion on coffee [16]. For medical students in MENA, over 98% drank caffeine-containing beverages in Jordan [2], and 72.3% drank coffee alone in Saudi Arabia [3].

Since the medical students at the University of Tabuk, KSA, are exposed to an overwhelming study schedule, as are students in other medical colleges, this may make them more likely to consume more caffeinated products, which would raise their susceptibility to the anxiogenic effects of caffeine. Additionally, to date, there is a shortage of data regarding medical students’ caffeine consumption habits and their relationship with mental health at the University of Tabuk, KSA. This study was conducted to assess the prevalence of caffeine consumption among medical students at the University of Tabuk, KSA, the level of awareness about the health effects of caffeine, and their mental well-being. Evaluating medical students’ awareness about the risks of drinking caffeine and their mental health issues will add to our understanding of the potential association between caffeine intake and mental health and increase the possibility of developing customized interventions to support mental well-being and academic achievement. This will be reflected in helping the students to choose healthier lifestyle options to promote their mental well-being.

## Subjects and methods

### Study design, setting, and timing

This cross-sectional research was conducted at the Faculty of Medicine of the University of Tabuk in Saudi Arabia over three months, from November 2024 until the end of January 2025.

### Study participants, sample size, and sampling technique

All medical students from the second through sixth academic years at the University of Tabuk were eligible to join the study. The Open Epi online calculator, version 3, was utilized to determine the sample size. With a total of 1015 students, 80% power, a 95% confidence interval, and a prevalence of 45.3% among medical students who consume caffeinated beverages daily, as per a previous study conducted in Saudi Arabia [3], the calculated sample size was 278 medical students. With an additional 10% added for non-response, the total number of medical students was 305.

A stratified random sampling technique with equal allocation was employed to include the students, with the stratification based on the medical students’ year of study. The eligible students in each academic year were selected using a computerized random selection technique from the students’ lists. A self-administered online questionnaire was disseminated electronically via the official university emails to minimize direct researcher-student interaction. To further minimize peer influence, the distribution process was supervised by the research investigators rather than the medical students. These steps have been undertaken to reduce selection bias and response bias.

### Data collection

An online self-administered Google form questionnaire, derived from a previous similar study [3], was disseminated. There were four parts to the questionnaire. The first part asked about the students’ age, gender, academic year, height, weight, and overall character. Caffeine consumption was the subject of the second section. Arabic coffee, brewed black coffee, black tea, soft drinks, and energy drinks were the various caffeinated beverages whose use and their frequency of intake were assessed, expressed in cups per day, and the associated factors for caffeine consumption.

A typical serving size for caffeine consumption is 237 milliliters, which contains 96 milligrams of caffeine in a cup of brewed coffee, 48 milligrams in a cup of tea, 33 milligrams in a can of soft drinks, and 79 milliliters in a can of energy drink [17]. The typical cup size for Arabic coffee is 25 ml, and each cup has roughly 4.0 mg of caffeine [18]. Each student’s daily caffeine intake was determined by multiplying the amount of caffeine in the caffeinated beverage they drank by the number of servings they consumed per day. For students who consumed multiple types of beverages, the sum of total caffeine intake from all types was calculated.

Regarding the third section it consisted of 11 questions assessing the students’ awareness about the effects of caffeine on their health, such as whether caffeine increases hours of wakefulness, diminishes the quantity and quality of sleep, raises heart rate, elevates blood pressure, induces heart palpitations, increased risk of heart disease, generates anxiety, brings about changes in body weight, causes digestive complications, affects food cravings, and serves as a diuretic, and possiblity of causing dehydration. The responses of the participants were either yes (scoring 2), no, or I don’t know (scoring 1). The total awareness scores ranged from 11 to 22, with a cutoff point established at 16.5, which represents the median value of the participants’ scores to discern those who were inadequately aware (below 16.5) from those who were adequately aware (16.5 or above). The concluding question in this section inquired if they had experienced any adverse side effects from caffeine consumption, with the respondents’ answers being either yes or no. Cronbach’s coefficient for this section was calculated to be 0.78.

Lastly, the fourth section employed the World Health Organization’s Five Well-Being Index (WHO-5) to assess mental well-being [19]. It’s a straightforward self-reporting tool containing five statements that reflect feelings from the past two weeks. Each statement is evaluated on a 6-point scale, with “all the time” (scoring 5) and “at no time” (scoring 0) serving as the extremes. This results in a total raw score for all questions ranging from 0 to 25. The raw score is converted into a percentage score between 0 and 100 by multiplying it by 4. Higher scores indicate enhanced mental well-being. The cutoff point is fifty percent to categorize this score into good (≥ 50%) or poor (< 50%).

Before the practical phase of the research commenced, a pilot study was executed involving twenty students who were excluded from the final sample and subsequent statistical analysis. The necessary adjustments were implemented on the questionnaire to enhance its clarity, brevity, and simplicity.

### Ethical consideration

All procedures performed in studies involving human participants were following the ethical standards of the institutional and/or national research committee and with the 1964 Helsinki declaration and its later amendments or comparable ethical standards. Ethical approval was secured from the Local Research Ethics Committee (LREC) at the University of Tabuk, Tabuk, Saudi Arabia, under the reference number (UT-434-244-2024). Data collection was performed using an anonymous online questionnaire; the initial page of the Google form questionnaire requests informed written consent from every participant; it contains a description of the study’s purpose and an affirmative statement regarding the confidentiality of the collected information. The participants must give their consent to proceed with the questionnaire.

### Data management and analysis

The data analyses were conducted utilizing Statistical Packages for Social Sciences (SPSS, IBM Corp, Armonk, USA) version 23. Descriptive statistics were presented using numbers and proportions, along with mean and standard deviation (SD). Body mass index (BMI) was calculated by dividing weight in kilograms by the square of height in meters. A chi-square test was performed to examine the association between categorical variables, while an independent sample t-test was utilized for testing the difference between the normally distributed continuous variables of two groups. The Spearman rank correlation coefficient (r) was calculated to assess the correlation between the levels of daily caffeine consumption, awareness of its side effects, and the WHO mental well-being score. To determine the factors predicting individuals’ caffeine intake, binary logistic regression was employed. A statistically significant outcome was established as a p-value of less than 0.05.

## Results

**Fig 1:**
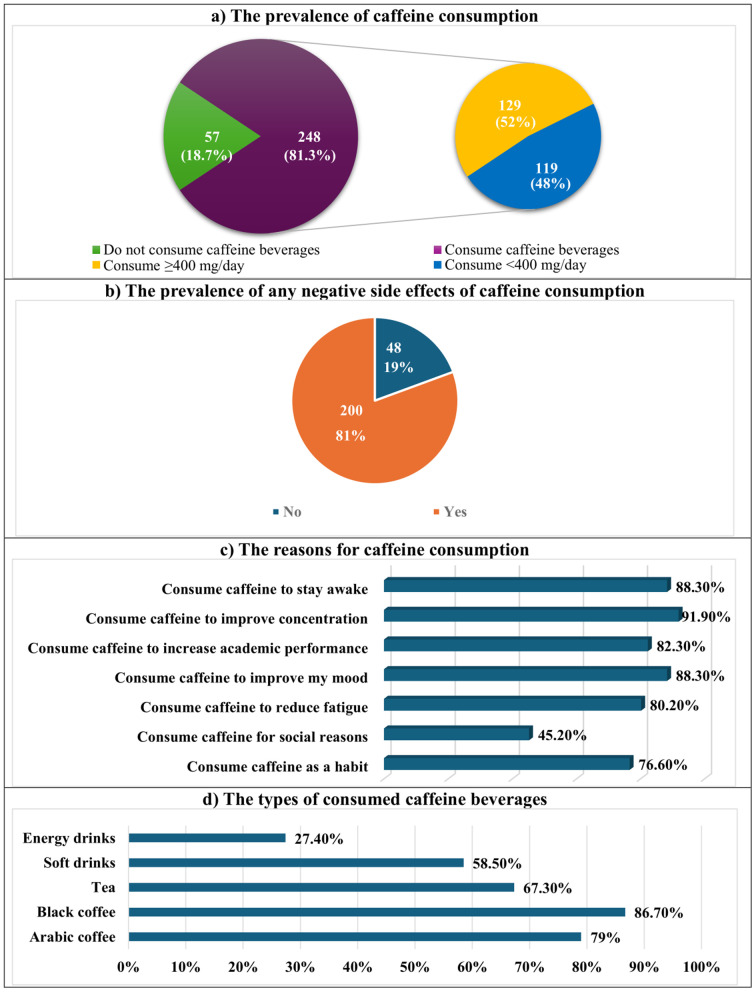
The prevalence of caffeine consumption, its side effects, and reasons for caffeine consumption among the studied medical students (n = 305).

Three hundred and five medical students, randomly chosen from each academic year from the second to the sixth, participated in this study. They were 52.8% male and ranged in age from 19 to 28 years, with a mean ± SD (21.3 ± 1.7). Out of them, 248 (81.3%) drank caffeinated beverages. Of the caffeine-consuming students, 52% had a high caffeine intake (≥ 400 mg/day) (Fig 1a). Two hundred medical students (80.6%) experienced the negative effects of excessive caffeine consumption (Fig 1b). Caffeine was consumed by the medical students in the study for a variety of reasons, including staying awake (88.3%), improving concentration (91.9%), increasing academic performance (82.3%), enhancing mood (88.3%), reducing fatigue (80.2%), social reasons (45.2%), or habit (76.6%) (Fig 1c). Black and Arabic coffee accounted for 86.7% and 79% of the caffeine-containing beverages consumed by the medical students under study, followed by tea and soft drinks (67.3% and 58.5%) and energy drinks (27.4%) (Fig 1d).

**Fig 2:**
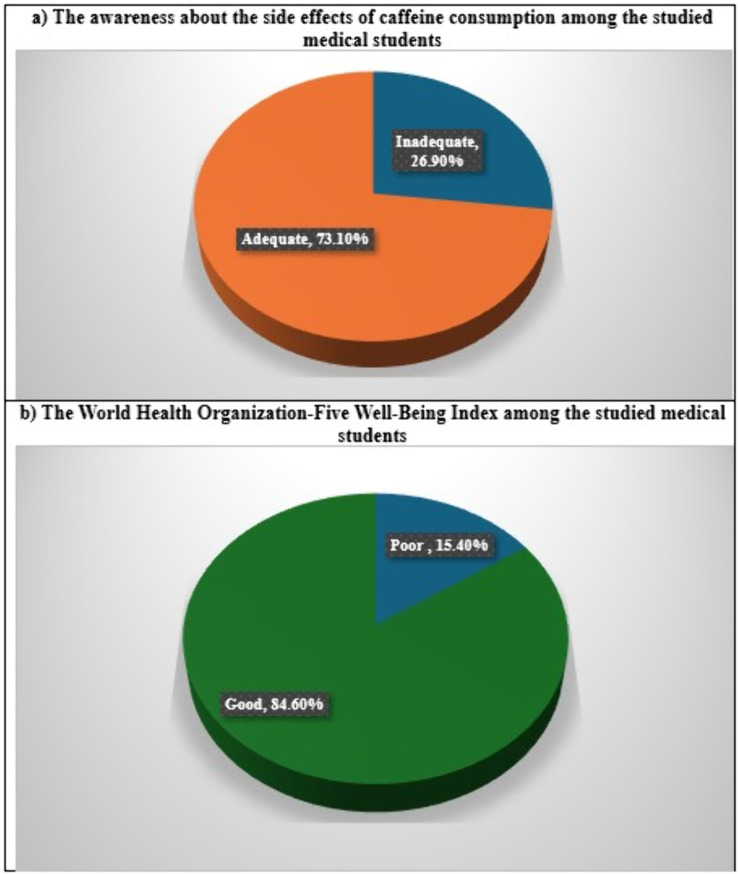
The prevalence of awareness about the side effects of caffeine consumption and the World Health Organization-Five Well-Being Index among the studied medical students (n = 305).

Of the medical students who were evaluated, 73.1% had an adequate level of awareness regarding the negative consequences of caffeine consumption (Fig 2a), and 84.6% had a good World Health Organization-Five Well-Being Index (Fig 2b).

Caffeine consumers had a substantially higher BMI (25.1 ± 6.1) than non-consumers (22.9 ± 3.9) (P = 0.01). Those who abstained from caffeine usage were more aware of its negative consequences than those who did (86.0% versus 70.2%, P = 0.01). The medical students who did not use caffeine had a better score on the WHO-Five Well-Being Index than those who did (100% versus 81%, P < 0.001) (Table 1).

**Table 1:** The association between caffeine consumption with sociodemographic characteristics and other parameters among the studied medical students (n = 305).

Parameter	Total studied medical students (n = 305)	The caffeine consumption		P value
		Yes (n = 248)	No (n = 57)	
**Age (year) mean ± SD**	21.3 ± 1.7	21.3 ± 2.1	21.3 ± 1.6	0.94 ^$^
**Gender**				0.98
Male	161 (52.8%)	131 (52.8%)	30 (52.6%)	
Female	144 (47.2%)	117 (47.2%)	27 (47.4%)	
**Academic year**				0.72
Second	61 (20.0%)	46 (18.5%)	15 (26.3%)	
Third	61 (20.0%)	50 (20.2%)	11 (19.3%)	
Fourth	61 (20.0%)	52 (21.0%)	9 (15.8%)	
Fifth	61 (20.0%)	50 (20.2%)	11 (19.3%)	
Sixth	61 (20.0%)	50 (20.2%)	11 (19.3%)	
**BMI: mean ± SD**	24.7 ± 5.8	25.1 ± 6.1	22.9 ± 3.9	0.01* ^$^
**The level of awareness about the side effects of caffeine consumption**				0.01*
Inadequate	82 (26.9%)	74 (29.8%)	8 (14.0%)	
Adequate	223 (73.1%)	174 (70.2%)	49 (86.0%)	
**The World Health Organization-Five Well-Being Index (%)**				<0.001*
Poor	47 (15.4%)	47 (19.0%)	0 (0.0%)	
Good	258 (84.6%)	201 (81.0%)	57 (100%)	

SD : standard deviation , BMI : Body Mass Index , * Significant difference.

Chi-square test was calculated. $ independent sample t-test was computed.

When comparing medical students who used less than 400 mg of caffeine per day, the majority of those who consumed more than 400 mg per day were male (69.8%) and in their fourth academic year (32.6%) (P < 0.001). The study found that 37.2% of medical students with high caffeine intake versus 21.8% of those with low caffeine intake were not sufficiently aware of the negative consequences of caffeine use (P = 0.008). 33.3% of the medical students in the study who consumed a lot of caffeine were more likely to have a bad World Health Organization-Five Well-Being Index, whereas 3.4% of those who consumed little caffeine tended to have a poor one (P < 0.001) (Table 2).

**Table 2:** The association between the level of caffeine consumption with sociodemographic characteristics and other parameters among the studied medical students who consume caffeine beverages (n = 248).

Parameter	The level of caffeine consumption		P value
	High intake ≥400 mg/day (n = 129)	Low intake < 400 mg/day (n = 119)	
**Age (year): mean ± SD**	21.2 ± 1.5	21.5 ± 1.8	0.07 ^**$**^
**Gender** Male	90 (69.8%)	41 (34.5%)	<0.001*
Female	39 (30.2%)	78 (65.5%)	
**Academic year**			
Second	26 (20.2%)	20 (16.8%)	<0.001*
Third	21 (16.3%)	29 (24.4%)	
Fourth	42 (32.6%)	10 (8.4%)	
Fifth	20 (15.5%)	30 (25.2%)	
Sixth	20 (15.5%)	30 (25.2%)	
**BMI: mean ± SD**	24.8 ± 5.3	25.4 ± 6.8	0.46 ^**$**^
**The level of awareness about the side effects of caffeine consumption**			0.008*
Inadequate	48 (37.2%)	26 (21.8%)	
Adequate	81 (62.8%)	93 (78.2%)	
**The World Health Organization-Five Well-Being Index (%)**			<0.001*
Poor	43 (33.3%)	4 (3.4%)	
Good	86 (66.7%)	115 (96.6%)	
**Consume caffeine beverages to stay awake**			0.005*
Yes	121 (93.8%)	98 (82.4%)	
No	8 (6.2%)	21 (17.6%)	
**Consume caffeine beverages to improve concentration**			0.45
Yes	117 (90.7%)	111 (93.3%)	
No	12 (9.3%)	8 (6.7%)	
**Consume caffeine beverages to increase academic performance**			0.02*
Yes	113 (87.6%)	91 (76.5%)	
No	16 (12.4%)	28 (23.5%)	
**Consume caffeine beverages to improve my mood**			0.005*
Yes	121 (93.8%)	98 (82.4%)	
No	8 (6.2%)	21 (17.6%)	
**Consume caffeine beverages to reduce fatigue**			0.15
**Yes**	108 (83.7%)	91 (76.5%)	
**No**	21 (16.3%)	28 (23.5%)	
**Consume caffeine beverages for social reasons** Yes	52 (40.3%)	60 (50.4%)	0.11
No	77 (59.7%)	59 (49.6%)	
**Consume caffeine beverages as a habit**			<0.001*
Yes	114 (88.4%)	76 (63.9%)	
No	15 (11.6%)	43 (36.1%)	
**Having any negative side effects from caffeine consumption**			
Yes	118 (91.5%)	82 (68.9%)	<0.001*
No	11 (8.5%)	37 (31.1%)	

SD : standard deviation , BMI : Body Mass Index , * Significant difference.

Chi-square test was calculated. $ independent sample t-test was computed.

Medical students with high caffeine intake were significantly more likely than those with low intake to think of reasons for their caffeine consumption, such as staying awake (93.8% versus 82.4%, P = 0.005), improving mood (93.8% versus 82.4%, P = 0.005), strengthening academic performance (87.6% versus 76.5%, P = 0.02), or as a habit (88.4% versus 63.9%, P < 0.001). Additionally, research showed that medical students who consumed high caffeine tended to have greater adverse effects than those who consumed less (91.5% versus 68.9%, < 0.001) (Table 2).

Male students (57.8%) and those in clinical academic years had an adequate awareness of the negative consequences of caffeine consumption compared to female students (42.2%) (P = 0.004), and those in basic academic years (P = 0.01). Furthermore, good mental well-being (90.1%) was most closely associated with an adequate level of awareness (P < 0.001). This was made clear after adjusting the other variables using binary logistic regression to predict adequate knowledge. The adjusted odds ratios for being male, in the sixth year, and having good mental well-being were 2.01 with 95% CI: 1.18–3.39, 3.73 with 95% CI: 1.5–9.27, and 3.94 with 95% CI: 2.07–7.53, respectively (Table 3).

**Table 3:** The association between the level of awareness about the side effects of caffeine consumption and other parameters among the studied medical students (n = 305).

Parameter	The level of awareness about the side effects of caffeine consumption		P value	Adjusted OR (95% CI)
	Inadequate (n = 82)	Adequate (n = 223)		
**Age (year): mean ± SD**	21.1 ± 1.4	21.5 ± 1.8	0.06 ^**$**^	1.12 (0.95-1.31)
**Gender**				
Male	32 (39.0%)	129 (57.8%)	0.004*	2.01 (1.18-3.39)*
Female	50 (61.0%)	94 (42.2%)		–
**Academic year**				
Second	22 (26.8%)	39 (17.5%)		–
Third	13 (15.9%)	48 (21.5%)		2.8 (0.93-4.66)
Fourth	16 (19.5%)	45 (20.2%)	0.01*	1.5 (0.73-3.43)
Fifth	23 (28.0%)	38 (17.0%)		0.93 (0.44-1.94)
Sixth	8 (9.8%)	53 (23.8%)		3.73 (1.5-9.27)*
**BMI:**	24.2 ± 5.2	24.9 ± 6.1	0.34 ^**$**^	1.01 (0.97-1.06)
**The World Health Organization-Five Well-Being Index (%)**				
Poor	25 (30.5%)	22 (9.9%)	<0.001*	–
Good	57 (69.5%)	201 (90.1%)		3.94 (2.07-7.53)*

SD: standard deviation, BMI: Body Mass Index, * Significant difference.

Chi-square test was calculated. $ independent sample t-test was computed.

Binary logistic regression was conducted, where the potential risk factors, such as gender, academic year, awareness level, and purpose of caffeine consumption, were simultaneously entered into the regression model. After adjusting for the effects of the other variables in the model, the adjusted odds ratios show the relationship between each factor and excessive caffeine use. The associated risk factors for excessive caffeine consumption were male gender (OR with 95% CI: 10.6, 3.8–29.3, P < 0.001) and the use of caffeine for a variety of purposes, including improving academic performance (OR with 95% CI: 8.4, 2.2–31.5, P = 0.002), staying awake (OR with 95% CI: 6.5, 1.4–29.4, P = 0.01), a habit (OR with 95% CI: 4.6, 1.9–11.4, P = 0.001), or enhancing mood (OR with 95% CI: 3.0, 1.4–6.5, P = 0.004). However, being highly aware of its adverse effects is protective (OR with 95% CI: 0.69, 0.58–0.82, P < 0.001). R^2^ was 0.45, and adjusted R^2^ was 0.44 (Table 4).

**Table 4:** Binary logistic regression to measure the predictors for high intake of caffeine consumption among the studied medical students who consume caffeine beverages (n = 248).

Parameter	β Beta	Adjusted OR (95% CI)	P value
**Gender** Male Female (reference)	2.3	10.6 (3.8-29.3)	<0.001*
**Academic year**			
Second (reference)			
Third	−0.44	0.65 (0.19-2.1)	0.47
Fourth	1.4	3.9 (0.79-19.2)	0.09
Fifth	1.1	2.9 (0.37-24.3)	0.30
Sixth	0.86	2.4 (0.15-37.7)	0.54
**The level of awareness about the side effects of caffeine consumption**	−0.37	0.69 (0.58-0.82)	<0.001*
**Consume caffeine beverages to stay awake**			
Yes	1.9	6.5 (1.4-29.4)	0.01*
No (reference)			
**Consume caffeine beverages to increase academic performance**			
Yes	2.1	8.4 (2.2-31.5)	0.002*
No (reference)			
**Consume caffeine beverages to improve my mood**			
Yes No (reference)	1.1	3.0 (1.4-6.5)	0.004*
**Consume caffeine beverages as a habit**			
Yes	1.5	4.6 (1.9-11.4)	0.001*
No (reference)			

Gender, academic year, awareness level, and purpose of caffeine consumption were simultaneously entered into the regression model.

**β:** Beta, OR : odds ratio; CI : confidence interval * Significant difference.

R² is 0.45, adjusted R² is 0.44.

**Fig 3:**
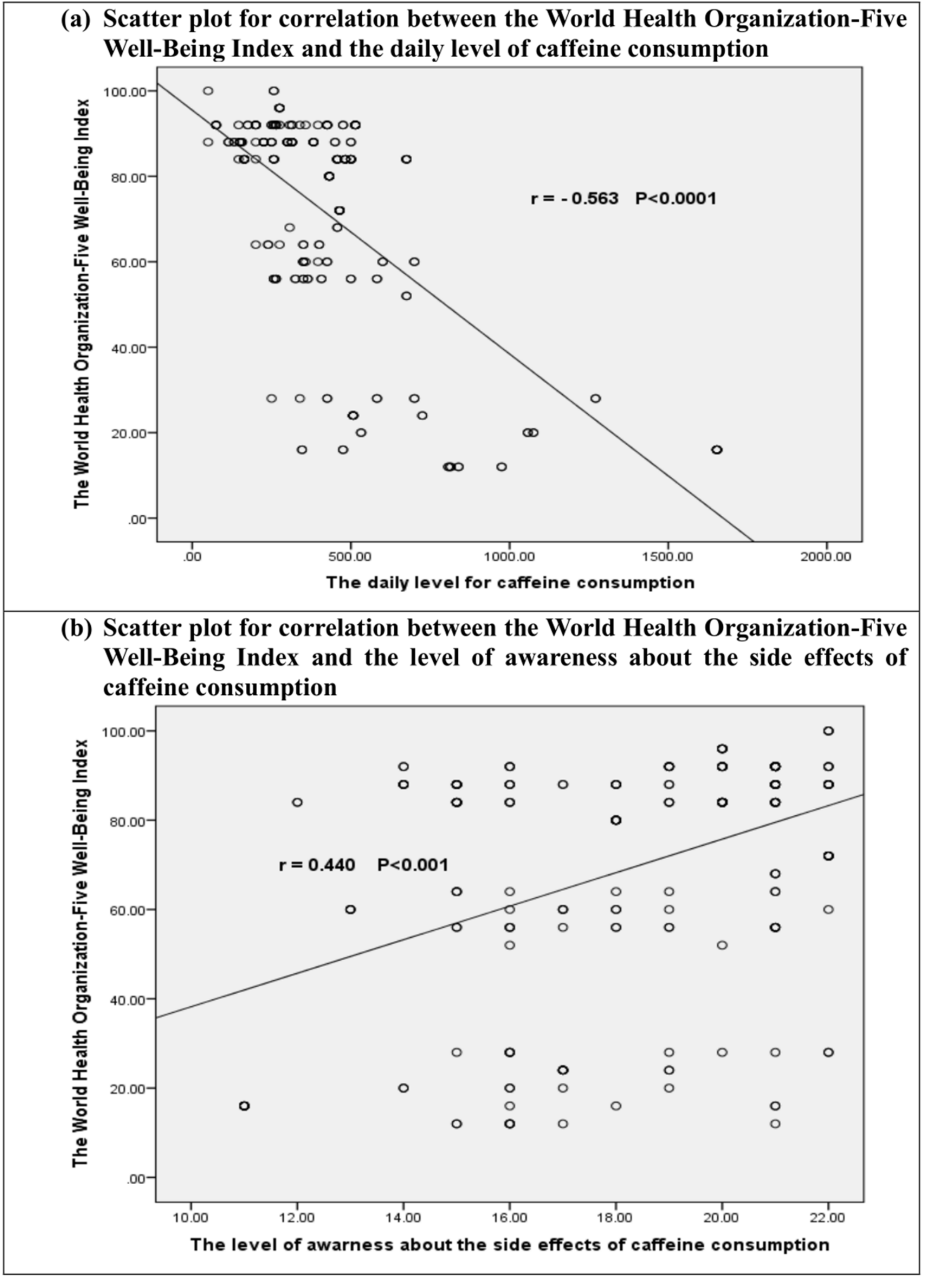
Scatter plot for correlation between the World Health Organization-Five Well-Being Index with the daily level of caffeine consumption and the level of awareness about the side effects of caffeine consumption. **r: Spearman correlation coefficient**.

Furthermore, the mental well-being score was significantly positively correlated with awareness level (r = 0.440, P < 0.001) (Fig 3a) and significantly inversely correlated with caffeine consumption level (r = −0.563, P < 0.0001) (Fig 3b).

## Discussion

Over the past few decades, many types of caffeine-based beverages have become a growing trend in Saudi Arabia and other Middle Eastern countries. Therefore, it was crucial to investigate the prevalence of caffeine consumption among University of Tabuk medical students and their awareness of the negative consequences of caffeine use as well as its implications for mental health.

About 81% of the students reported consuming caffeinated beverages. This high level of consumption was nearly identical to what was observed in earlier studies of a similar nature [11,20–22]. Black coffee and Arabic coffee were the most consumed caffeinated beverages among the medical students in our study, followed by tea, soft drinks, and at least energy drinks. This is similar to the results from studies conducted in Saudi Arabia [3,23], while different from a study conducted in Egypt [24], where soft drinks were the most consumed, followed by coffee and tea.

The high consumption among students may be explained by the fact that all forms of caffeinated beverages are readily available and reasonably priced, with no restrictions on their purchase or use. In addition, the academic burden on medical students, in particular, puts them under stress and makes them try to compete by drinking coffee.

There is constant discussion on the relationship between body weight and caffeine intake. The results of the current study showed that caffeine-consuming students were more likely to have higher BMIs than those who did not consume caffeine, which is consistent with previous research from Korea and Saudi Arabia [25–27]. This runs counter to earlier studies that found little or a negative correlation between coffee consumption and weight gain [4,28,29]. One of the primary causes of the inconsistent effects of caffeine on human health is variations in its composition and consumption habits, including the type and amount of caffeine consumed. Consuming caffeinated beverages and soft drinks with high sugar and calorie content might cause an increase in body weight. Furthermore, it was observed that excessive Arabic coffee intake is associated with higher levels of total and low-density lipoprotein cholesterol in the blood [30].

Compared to earlier studies conducted in Spain (9.5%) and Malaysia (2.6%) [21,31], most caffeine users in this study (52%) consume more than 400 mg of caffeine per day. Still, it closely resembles research done in Dubai (35%) and Saudi Arabia (43.1%), respectively [32,33]. Given that students’ first choice for caffeinated beverages is dark coffee, which has a high caffeine content, Arabian coffee, which came in second place in this study, is extracted from Coffea Arabica beans, which belong to the Rubiaceae plant. In Saudi Arabia, it is the most widely consumed hot beverage served in small cups and is deeply ingrained in Gulf nations. It is considered to be a form of hospitality that is offered regularly throughout the day and at any time. Arabian coffee contains significantly lower caffeine content than other caffeine beverages. The differences in findings between research could be explained by factors related to society and culture [29,34]. This is supported by the fact that 76.6% of the students in this survey reported consuming caffeine as a habit, with 88.4% of them consuming high caffeine amounts each day.

Moreover, staying awake, improving mood, and enhancing academic performance are among the additional factors that were significantly linked to and predicted excessive caffeine consumption. It could be recognized that all these reasons had a connection to the student’s academic and educational purposes. The reasons given for caffeine consumption are consistent with those found in earlier research, including students in Korea, New Zealand, and Spain [12,20,21]. Given the potential negative effects of high caffeine usage, future studies and strong efforts should be directed at these heavy users to prevent any health issues.

It should be highlighted that during times of higher academic stress, such as exam season, patterns of caffeine usage and the reasons for them may vary throughout the academic year. According to one study, 63% of college students report drinking more coffee at test time [12].

Excessive caffeine use, more than permitted, can lead to caffeine intoxication, also known as caffeineism. Anxiety, cardiovascular symptoms, insomnia, and gastrointestinal problems are all associated with it [35]. Most of the medical students in the study who drank caffeinated beverages reported experiencing harmful side effects from their caffeine intake, especially among those with high caffeine intake per day. This may be explained by the fact that over half of the students consume more than 400 mg of caffeine every day. Similar studies found that excessive daily caffeine use was associated with the emergence of negative effects [24,36].

Most students are adequately aware of the negative effects of caffeine use. Those who avoided drinking caffeine or drank less than 400 mg per day were likely having a greater level of adequate awareness, which is comparable to a KSA study [26]. Students in clinical years (4th, 5th, and 6th years) had significantly greater awareness about caffeine side effects than those in basic years; this could be explained by their accumulated greater scientific and medical knowledge from exposure to clinical courses during their study. This adequate awareness was associated with their low intake of caffeine per day, except for fourth-year students, who were found to consume high daily levels. One explanation could be that this year marks the transition from the academic years to the clinical years at the University of Tabuk’s Faculty of Medicine. Due to their new course load and altered study environment, students may become more prone to stress, which could result in a variety of coping mechanisms, including increased coffee consumption.

The male students in this study are adequately aware of the adverse effects of caffeine; this is similar to the results of an Indian study, which concluded that male students are more knowledgeable about caffeine side effects than female students [37]. Despite this, it has been noted that males consume a notably high level of caffeine. This is similar to other research in Korea and Saudi Arabia [12,16,23,38], but it differs from studies in the USA and Spain, where females consumed more [11,21]. These results suggest that high awareness may not be sufficient to change the practice and that several factors other than knowledge may prevent healthy behavior, including cultural expectations, daily lifestyle, and the need for caffeine-stimulating effects, may influence males’ increased caffeine consumption relative to females. Females, on the other hand, might prefer lower doses of coffee for its emotional and social benefits rather than merely for its stimulating qualities. Furthermore, it is noteworthy that the biological difference between both genders is represented in the greater detrimental effects of caffeine on females than on males [16].

Caffeine is mostly antagonistic to adenosine A₁/A₂A receptors, which can significantly increase dopamine and norepinephrine release, improving mood and alertness. However, side effects like anxiety, sleeplessness, and an increased heart rate can occur with high dosages [39]. Exposure to stressful academic life for medical students makes assessing their mental well-being crucial. About 85% of the studied medical students had good mental well-being. This is higher than that recorded from a similar study in Hungary (36.4%) [40].

The impact of caffeine on mental health has generated much discussion and contradictory research findings. The results of this research demonstrate a significant negative correlation between mental health and caffeine consumption. All students who abstained from caffeine and consumed less than 400 mg per day were more likely to have better mental health, as small doses of caffeine are believed to reduce anxiety and enhance mood [41], while excessive coffee drinking has been linked to increased stress with considerable irritation and confirmed poor mental well-being [42]. This is contrary to the findings of earlier research in Saudi Arabia and Malaysia, which found no significant association between students’ mental well-being and caffeine consumption [23,31]. The results of the current study show a significant positive correlation between students’ mental health and their awareness of the negative effects of caffeine use. This emphasizes the importance of informing students about caffeine consumption and its long-term effects on their physical and mental health.

### Strengths and limitations

This study is the first of its type in Saudi Arabia and the University of Tabuk to evaluate medical students’ overall mental well-being and its relationship to caffeine use using the World Health Organization’s Five Well-Being Index which is considered a valid and reliable screening index for mental well-being, as it reported that 52% of the respondents consumed ≥400 mg of caffeine daily and to find a moderate to high negative correlation with WHO – 5 scores (r = –0.563, P < 0.001)

It is essential to acknowledge this study’s limitations. The current study was limited to cross-sectional self-reported data from students, which may be subject to recall bias. In addition to the fact that the cause-and-effect relationship could not be exactly confirmed from this study design. Since the amount of caffeine consumed throughout exam periods varies, as a result, it is possible that the results here do not fairly reflect caffeine consumption during the whole academic year. Also, to a large extent, the caffeine intake in this study was mainly from coffee. Besides caffeine, coffee is also rich in various bioactive components, such as caffeoylquinic acids, caffeic acid, and quinic acid. These substances have antioxidant, anti – inflammatory, and neuromodulatory effects and may have independent or synergistic impacts on mental health [43]. Therefore, this study cannot distinguish the pure effect of caffeine from the combined effects of other components.

### Practical implication

The study’s practical implications include providing valuable insights into the awareness, mental health, and caffeine intake patterns of medical students. It’s crucial to conduct health education programs to increase the medical students’ awareness about the negative effects associated with caffeine use and improve their consumption habits. The availability of student wellness programs to support the mental well-being of the students, including suggestions for caffeine use to decrease its adverse effects on mental health, is required. Also, the results of this study can help universities to substitute the high-caffeinated beverages with healthier options. To analyze the potential mechanisms of each coffee component, assess the causal effect relationship, and highlight the detrimental effects of prolonged caffeine use, future longitudinal studies, clinical trials, and community-based research will need to be conducted. This can assist in developing evidence-based guidelines for the consumption and promotion of caffeinated beverages to the public and medical students.

## Conclusion

Caffeine consumption is remarkably prevalent among medical students at the University of Tabuk. More than half of the students consume more than 400 mg of caffeine per day; being male, having inadequate awareness, wanting to stay awake, improved mood, increased academic performance, and a habit are significant associated factors for the high level of consumption. The majority of the students have good mental well-being, which is negatively correlated with high caffeine consumption. Most of the participants have adequate awareness about the negative side effects of caffeine, which was positively correlated with their mental well-being. It is recommended that university students take part in caffeine literacy education programs and update their educational curriculum to increase their awareness of the adverse physiological consequences of consuming significant quantities of caffeine-containing foods and beverages.
